# Perceived Facilitators and Barriers to Participating in a Combination Income-Generating HIV Risk-Reduction Intervention Among Adolescent Girls and Young Women in Nigeria: A Qualitative Study

**DOI:** 10.3389/frph.2021.560908

**Published:** 2021-12-13

**Authors:** Ucheoma Nwaozuru, Wakilat Tijani, Titi Gbajabiamila, Chisom Obiezu-Umeh, Florida Uzoaru, Oliver Ezechi, Adesola Z. Musa, Jami Curley, Rhonda BeLue, Juliet Iwelunmor

**Affiliations:** ^1^Department of Behavioral Science and Health Education, College for Public Health and Social Justice, Saint Louis University, Saint Louis, MO, United States; ^2^Nigerian Institute of Medical Research, Lagos, Nigeria

**Keywords:** HIV, risk-reduction, adolescent girls and young women, Nigeria, income-generating intervention

## Abstract

**Background:** Human immunodeficiency virus risk-reduction interventions that include income-generating activities are garnering attention as effective strategies to engage adolescent girls and young women (AGYW) toward HIV risk reduction. To sustain and promote the uptake of these interventions, researchers must understand factors that may encourage or present barriers to AGYW participation in such interventions. This study explores AGYW perceived barriers and facilitators to participation in a school-based combination income-generating HIV prevention intervention in Nigeria.

**Methods:** A convenience sample of AGYW who participated in a school-based combination income-generating HIV prevention intervention were recruited for the study. Data generated from focus group discussions (FGDs) (eight discussion groups comprising 10–12 participants) were analyzed by inductive thematic analysis.

**Results:** A total of 93 participants with a mean age of 15.04 years (SD = 0.89) participated in the FGDs. The study participants identified several facilitators and barriers to participation in the intervention. Three main themes that emerged as facilitators were: (1) involvement of young female facilitators in the delivery of intervention components, (2) opportunity for social interaction with peers during the intervention period, and (3) support and approval from school authorities. Two main themes were also identified as barriers: (1) sexual conservatism from society and parents and (2) challenges in sustaining a microenterprise.

**Conclusions:** Despite the perceived benefits and interest in participation in the intervention, the study participants outlined some challenges that may hinder participation in the intervention components. Addressing barriers, such as stigma associated with the discussion of sexual health-related topics, coupled with the promotion of facilitating factors, such as leveraging context-appropriate intervention delivery modalities, is important for enhancing the engagement of AGYW in HIV risk-reduction intervention. Our findings can guide future research and design of combination income-generating HIV prevention interventions for in-school AGYW in low-resource settings such as Nigeria.

## Introduction

The incidence of HIV is declining in many settings. However, rates of new HIV infections remain high among adolescent girls and young women (AGYW) aged 15–24 years in sub-Saharan Africa (SSA) (1–3). In Nigeria, AGYW continue to account for a disproportionately higher number of new HIV diagnoses (4, 5), where heterosexual sex is the dominant mode of transmission. AGYW in Nigeria are more than thrice as likely to be living with HIV than their male peers, with an HIV prevalence rate of 1.9% among females vs. 1.9% prevalence rate among males (4, 5). The disproportionate burden of HIV among AGYW reflects age–sex disparate relationships, in which HIV is mainly transmitted inter-generationally from an adult male to adolescent female sexual partners, and transactional sex, where young women depend on older men financially (6, 7). Such relationships are prone to power imbalance, decreasing the agency and ability of women to practice safe sex, increasing their risk of HIV infections. In response to the high rate of HIV infections among AGYW, numerous HIV interventions exist that address factors that increase their vulnerabilities to HIV. These include interventions focused on decreasing transactional sex, promoting safe sex, promoting agency, and safe sex negotiation. Particularly, research on HIV prevention that combines economic strengthening components are increasingly recognized as effective strategies to engage AGYW toward HIV risk reduction (8, 9).

Combination HIV prevention interventions that include income-generating activities have shown promising results in HIV risk reduction intentions and behaviors among AGYW in SSA (10, 11). Income-generating components include microfinance, vocational skills training, business development training, microenterprise development, cash transfers, and savings-led asset-based programs that work to alleviate the economic hardship of AGYW by providing skills to allow them to generate economic assets and resources (12–14). While the benefits of implementing such interventions have been established, there is a need to actively engage and reach the target population for the interventions to be impactful. Therefore, researchers must understand factors that may encourage or present barriers to the participation of AGYW in these interventions to promote and sustain the reach and uptake of these interventions. Despite the need to understand these factors, many intervention studies are mainly concerned with assessing the effectiveness of the intervention, with limited focus on factors that influence participation in the intervention.

We implemented a combination income-generating HIV prevention intervention among in-school adolescent girls in Nigeria. One hundred and twenty-seven (127) AGYW attending three secondary schools were assigned to the intervention condition, and 178 adolescent girls in three schools were assigned to the control condition. This study describes perceptions of the adolescent girls who participated in the intervention arm of the study. The specific objective of the study was to explore experiences of the study participants, as well as perceived barriers and facilitators to participation in a combination income-generating HIV prevention intervention in Nigeria.

## Methods

### Study Setting and Participants

This study was cross-sectional and was conducted among students attending secondary schools in Lagos State, Nigeria. Lagos State is situated in the Southwest region of Nigeria, bordered by the Atlantic Ocean. It is the most populous city in Africa, with an estimated population of 17 million people out of a national estimate of 150 million in Nigeria (15). The state comprises individuals from diverse ethnic backgrounds of Nigeria and an ideal location to implement the intervention, including consideration of the local culture, population, and context to ensure that programs are adapted to fit the needs of consumers. This study consisted of adolescents attending three secondary schools in the state who participated in a combination income-generating HIV prevention intervention from June 2019 to August 2019.

### Intervention

The intervention consisted of four 2-h interactive group sessions implemented at the intervention schools. Asset theory (9) and social cognitive theory (16, 17) were complementary theories that guided the design and implementation of the intervention. The asset theory suggests that having a source of economic stability can improve the expectations of AGYW for the future, leading to positive health behaviors, such as attitudes toward not engaging in HIV risk behaviors (9). The social cognitive theory emphasizes that the agency of an individual to adopt HIV prevention-related behavior is partly shaped by their sociocultural environment (16, 17), such as their knowledge of economic conditions, goal-setting, and decision-making autonomy (18). The components of the intervention were focused on enhancing the knowledge and financial skills of participants, and resources to develop agency for HIV risk reduction.

The four-session combination income-generating HIV prevention intervention included: (1) HIV prevention education: the objective of the health education sessions was to improve the knowledge and understanding of HIV and foster self-efficacy, communication skills, and risk reduction strategies; (2) income-generating activity (i.e., microenterprise training for jewelry-making, and marketing): the objective of the enterprise training sessions was to provide entrepreneurial and business training skills to the participants; (3) youth development funds (YDFs): the objective of this component was to provide asset accumulation to the participants and to support the development of the jewelry microenterprise of the participants financially. All the participants in the intervention group received 1,000 naira (equivalent to $3) after the intervention to assist with purchasing materials for their jewelry microenterprise. This amount was decided based on prior interactions with stakeholders (students, teachers, and community leaders). Table 1 presents an overview of the intervention components. The study participants were eligible to participate in the study if they were 13 years and above, participated in the intervention, attended secondary schools, and enrolled in JSS1, JSS2, JSS3, SS1, SS2, and SS3 (equivalent to Grades 6 through 12 in the United States), and able to provide informed consent. According to the Nigerian guidelines for sexual and reproductive health research, young people who are 13 years and over can provide informed consent for sexual and reproductive health research.

**Table 1:** Intervention components.

**Intervention curriculum**		
	**HIV prevention education**	**Microenterprise training**
Session 1: Values	Introduction/Identification of values: girls prioritize their values, explore discrepancies with behaviors	Discuss the value of beads in the Nigerian context. Learn colors, textures, and materials for bead-making
Session 2: Knowledge and skills building	Gender-specific sexual and reproductive health education: enhance HIV/AIDS knowledge and perceived vulnerability, in the Nigerian context. Increase the ability to recognize and label risky behaviors. Enhance knowledge and skills in addressing menstrual pain, testing for HIV, PrEP services	Learning beaded jewelry-making techniques. Making of beading jewelry
Session 3: Communication and skills building	Problem-solving and communication skills: assertive, aggressive or passive; non-verbal. Safer sex skills, including condom use, abstinence, and resisting peer pressure	Beaded jewelry-making
		Communication and marketing skills/role-playing. Develop business vision and plan
Session 4: Risk-reduction/value creation	Developing strategies for preventing HIV and other STIs. Application of communication skills to develop safe sex negotiations. Learning about decision-making and goal-getting as strategies for HIV prevention behaviors. Designing sexual risk-reduction messaging to be included in jewelry	Design and package items for the sales event. Discuss the value of jewelry and value-based selling and pricing strategy
		Plan for sales event

### Study Design and Data Collection

We conducted eight focus group discussions among the 93 participants who completed the intervention. Each focus group discussion comprised 10–12 individuals who recruited through face-to-face announcements. We conveniently recruited students who had participated in the intervention to participate in the focus group discussions. No refusals occurred, and all the students present in school on the day of data collection were interested in participating in the focus group discussions. Focus group discussion leverages the interaction between participants to generate richer data than might be obtained from individual interviews (19). The focus group discussions were conducted by two research team members (UN, WT) who were also Nigerian women. One of the researchers (UN) led all the focus group discussions, and WT assisted by taking field notes during the data collection process. The two research team members (UN, WT) also facilitated the intervention and have previous experience in conducting sexual and reproductive health research, including qualitative data collection among young people in Nigeria. The focus group discussions were conducted in classrooms provided by the school authorities in the school premises.

Verbal informed consent was obtained from all the study participants prior to beginning the focus group discussions. The participants were informed about the research using an informational letter about the purpose of the study. Additionally, the purpose of the focus group discussion was explained before commencing the focus group discussions. Demographics of the study participants were collected during the focus group discussion. The discussions lasted between 45 and 60 minutes, and were moderated by two members of the research team (UN, WT) using a semi-structured guide with a series of probes to help stimulate conversation and generate additional information from the participants. The discussions centered on barriers and facilitators of participation in the intervention. The questions included topics on factors that made it easier or difficult for them to participate in the intervention. We also asked the participants to share their overall experience with the intervention. Probing questions were utilized to extract further information and discussion among the study participants. With consent from the participants, we audio-recorded each focus group discussion.

### Data Analysis

Frequencies and proportions were calculated for the demographic characteristics of the participants. All the focus group discussions were transcribed verbatim into a word document. An inductive thematic analysis approach was used for analysis as described by Braun and Clarke (20). Given the exploratory nature of the focus group discussions, data analysis was without the presupposition of the existing theoretical framework (21), and data were manually analyzed following six sequential steps. This involved familiarization with transcripts, forming initial codes, searching for themes, reviewing themes, defining and naming themes, and producing reports. In our report, although the themes may have been influenced by the primary research questions, quotes from the focus group discussions were used to illustrate each important theme identified during data analysis. To assess the accuracy of our results, we debriefed the findings of the focus group discussions with the study participants. There were no changes made to the data following the debriefing. Given the consistency with the results and the perceptions of the participants, the COnsolidated criteria for REporting Qualitative (COREQ) research checklist was adhered to for study reporting, analysis, and interpretation (22); see Supplementary File 1.

### Ethical Approval

The study was approved by the Nigerian Institute of Medical Research Institutional Review Board (IRB/19/028) and the Saint Louis University Institutional Review Board (Assurance No: FWA00005304; Protocol No: 30338). Prior to participation in the study, all the participants provided informed consent.

## Results

### Description of Study Participants

Ninety-three participants took part in eight focus group discussions. All the participants completed the combination income-generating HIV prevention intervention. Most of the participants (45.2%) were in SS1, Christians (66.7%), and from the Yoruba ethnic group (74.2%). A larger proportion of the participants lived with their parents (91.4%), compared with those living with other relatives who were not their parents (8.6%). The characteristics of the participants are presented in Table 2.

**Table 2:** Characteristics of study participants (*N* = 93).

**Characteristics**	***n* (%)**
Mean age in years (SD)	15.04 (0.89)
School assignment	
School 1	21 (22.6)
School 2	48 (51.6)
School 3	24 (25.8)
Highest education level	
JSS2	5 (5.4)
JSS3	6 (6.5)
SS1	42 (45.2)
SS2	40 (43.0)
Type of school	
Government-owned	69 (74.2)
Private-owned	24 (25.8)
Ethnicity	
Yoruba	69 (74.2)
Igbo	18 (19.4)
Other ethnic groups	4 (4.3)
Hausa	2 (2.2)
Religion	
Christianity	62 (66.7)
Islam	31 (33.3)
Living arrangement	
Living with parents	85 (91.4)
Living with relatives, other than parents	8 (8.6)

### Facilitators of Participation in the Intervention

Three main facilitator themes were identified: (1) involving young female facilitators; (2) opportunity for social interaction with peers; and (3) support from the school authorities.

#### Involving Young Female Facilitators

The participants consistently identified that engaging young female facilitators was a major facilitator of participation in the intervention. The participants articulated strong views on the importance of involving female facilitators in sexual and reproductive health interventions for AGYW. It facilitated the approval of parents and guardians for their children/wards to participate in the intervention.

According to one of the participants,

“when I told my mother about this program, she asked me if is a man or a woman that will be teaching us. When I told her it will be a woman, she said it is okay for me to be attending the program. I am thinking maybe if it is man teaching us this type of topic from outside she will not like it and can tell me not to attend it” (SS2, 17 years).

Beyond receiving approval to participate in the intervention, the participants indicated that having female facilitators made them feel comfortable asking questions and actively engaging in the intervention activities. The female facilitators provided a safe space for the participants to share their concerns and ask questions freely. According to one of the participants, “*the aunties teaching us were very nice, and I think they understand us because they are also women. This made it easy for me to come back for the program everyday”* (JSS2, 14 years).

A number of the participants appreciated having conversations around sexual and reproductive health with the facilitators without the fear of being judged. One participant stated,

“the aunties were very calm with us, I feel very free to talk to them about anything. I even asked questions that I will be shying from before. For example, when I was asking about the meaning of anal sex, I was not feeling shy like before. They aunties made me feel very free and did not judge me. This made me come back to learn new things everything. I even learn about HIV self-test, I did not know about it before the program” (SS1, 16 years).

#### Opportunity for Social Interaction With Peers

Individuals who participated in the intervention appreciated the opportunity to interact with their peers in safe and youth-friendly space. This helped foster meaningful dialogues on social, cultural, and behavioral factors related to HIV prevention among AGYW. One of the participants expressed,

“I was coming back to the program because I can talk to my classmates freely here. Some of them, especially the juniors, I did not talk to them before now. But this program allow us to talk here because we are learning skills to prevent AIDS among young girls in Nigeria” (SS2, 16 years).

Another participant explained,

“I liked going to the program because I can meet with other girls to talk about our goals, even the boys in our school wanted to come to the program but we told them it is only for girls. So, this is a very good program because it let us learn in an enjoyable way. It is not like learning in the class, in this program we enjoy ourselves, talk with each other and also learn important things” (JSS3, 13 years).

#### Support From the School Authorities

The participants indicated that approval from the school authority made it easy for them to participate in the intervention. For the participating schools, the interventions were implemented during times allotted by the school authority to ensure that interested students who met the eligibility criteria were available to participate in the intervention.

One participant reported that, “*[b]ecause the program is in school my parents did not have problem telling me to attend it. I think if I had to go to a far place for this program my parents may have problem with it and will not be able to attend it. They trust that school can only approve a program that is good for us”* (SS2, 17 years).

### Barriers to Participation in the Intervention

Two main themes emerged as barriers to participation in the intervention. These included: (1) sexual conservatism from society and parents and (2) cost barriers with sustaining a micro-enterprise.

#### Sexual Conservatism From Society and Parents

In discussions on some barriers to participation in the intervention, the participants consistently mentioned discomfort discussing sexual and reproductive health issues in schools. Although a good number of the participants acknowledged that conducting the intervention in the school made it convenient and easy to obtain approval from their parents and guardians, it posed a barrier for some of their peers to participate in the intervention. One of the participants mentioned:

“some of us do not like talking about sex things in school, other students who are not part of the program may be looking at us somehow. This can discourage someone from coming to the program” (SS2, 18 years).

While commenting on the issue of discomfort with discussing sexual and reproductive health topics on the school premises, several participants mentioned that they found some of the questions in the survey to be uncomfortable. This was illustrated in statements such as:

“I am not having sex, so of the questions are not for me. It is for big big girls in university. I don't like answering that question. Some of the questions made me uncomfortable and was too long” (SS2, 17 years).

Another participant echoed similar sentiments by stating,

“Maybe you should have different questionnaires for young girls and bigger girls, those questions on sex in the questionnaire were not comfortable. I feel like my mother will not like me to answering that question because I am not having sex” (JSS3, 13 years).

While most of the participants considered the intervention component to be instrumental in building skills toward HIV prevention practices, some of the participants cited concerns that some of the topics on HIV prevention, such as condom use, may promote premarital sex, which is against their religious beliefs and moral values. One participant explained,

“you know since I do not want to have sex before I get married, hearing things like how to use condom, PrEP can make some people want to have sex. But this is bad because my mother and people in church tell us it is not good to have sex before we get married. I am a young girl and I am not having any sex until I get married” (SS1, 15 years).

When asked about how to address the concern of sexual conservations, some of the participants suggested having tailored intervention components for sexually active and non-sexually active individuals. Some of the participants believed that this would encourage participation among adolescent girls and adherence to intervention components.

#### Cost Barriers With Sustaining a Microenterprise

The emergent subtheme related to challenges with sustaining a jewelry microenterprise was consistently identified as a major barrier to participation. The participants expressed concern that the youth development funds provided at the completion of the intervention were not adequate to start a jewelry microenterprise successfully. As one participant said,

“I really wanted to make more beads but I did not have enough money to buy the beads. I wish I have more money I will make more” (JSS2, 14 years)

Some of the participants who were able to continue with their jewelry microenterprise emphasized the need for additional funds for continuity and encouraged future participation. One participant stated,

“The three bracelets I made I sold them for 250 naira, if I have more money I can make more more bracelets. People are asking me for more. I think it will also make more people to want to participate in this program because they know they will be making money to be self-reliant as girls. I can even continue making money when I enter university” (SS1, 15 years).

In addition, some of the participants mentioned that they could not source the materials to make the jewelries and did not have customers to purchase their products. According to one participant,

“I want to continue with this program, but my main problem is that I do not even know where to buy the things to make the beads. You know my parents do not allow me to just go to market. This will make me not want to continue because I cannot practice what I learned after the program” (SS2, 17 years).

In agreement with this, another participant stated,

“I have the same problem, I do not even know where to buy the materials to make the beads and I do not even know where to get customers. I only meet people in school and church. My parents do not allow me to go to places anyhow. So I do not even know how I can be making money with this. It is not encouraging me to be in this program” (JSS3, 14 years).

In line with challenges with building a microenterprise, a large proportion of the participants stated that providing only jewelry-making training may pose a challenge to engaging young girls in the interventions actively. Some of the participants explained that some of their schools already offer bead-making classes in their junior secondary school home economics classes. To address this, the participants suggested providing a variety of microenterprise training. This included options such as tailoring, tie-and-dye, shoe-making, gele-tying (head wrap), and make-up training. A variety of options would allow study participants to select vocational skills training that best meets their needs and interests. The participants further suggested that the intervention could include a component to assist the participants with securing customers for their microenterprise. As one participant mentioned,

“If they [the intervention facilitators] can teach us how to get customers and where to sell our things, it will make a lot of people want to be part of the program. The way the program is now, we have to find people to buy our things which is very very hard. This can be making some people not want to be coming for the program again” (SS1, 16 years).

### Participant Experience With the Intervention

Beyond barriers and facilitators of their participation in the intervention, we also asked the study participants to share their overall experience with the intervention. Two overarching themes emerged that sum up their experience: (1) intervention structure and components, and (2) skills acquisition.

#### Intervention Structure and Components

The participants generally found the intervention structure acceptable and appropriate. Most of the participants stated that they enjoyed the interactive and collaborative structure of the component. A participant stated that,

“my experience in the program was good, I liked that you did not just teach us. I really enjoyed it when we did the play [role-play]. It was different from learning in class, we were having fun and learning at the same time. The class was very interactive” (JSS2, 13 years).

Another participant also explained,

“I liked that we learned the HIV prevention and bead-making. I did not want the bead-making to end. It made it not be boring” (SS1, 15 years).

Regarding the most favorite component of the intervention, the majority of the participants shared that they liked the components on value identification, communication, jewelry-making, goal-setting activities, and role-plays the most. The least favorite component of the intervention among the majority of the participants was completing the study questionnaires. Most of the participants stated that the questionnaires were too lengthy. One of the participants stated, “I *did not like the questionnaires, we have too much questions”* (SS2, 16 years).

When further asked about the duration of the intervention, there were mixed responses among the participants. Some of the participants indicated that the length of the intervention was adequate to keep them actively engaged throughout the intervention sessions. This was highlighted in this statement, “*I think the time for the program is enough, I think if it is too long I would not like it and I can become boring”* (SS1, 14 years).

Another group stated that they would want the intervention to be 3 h, especially to provide more time for the jewelry-making component of the intervention. One participant said,

“I would want it [the intervention] to be 3 h, …, I want more time for the jewelry-making, so that you can teach more things and to give us time to practice more during the program. Some days, I did not have time to finish my bead when I go home” (SS2, 18 years).

#### Skills Acquisition

When describing their experience with the intervention, most of the participants discussed how their knowledge of HIV had improved. For instance, the majority of the participants were exposed to HIV self-test during the intervention. A participant mentioned that, “*I learned a lot of things on how to protect myself from HIV from this program. I did not even know that you can test for HIV by yourself. Now I want to do it”* (SS1, 15 years).

From this experience, many of the participants wanted to use the HIV self-test kit demonstrated during the intervention; however, we could not offer it to the participants because we did not have ethical approval for it, and it was beyond the scope of the intervention.

Some of the participants also shared how they were able to transfer some of the knowledge and skills acquired from the intervention to their family members and peers:

“I was teaching my mother and my sister about HIV self-testing and she was very proud of me. My mother was happy I was learning something from the program” (JSS 2, 13 years).

Another participant also shared,

“I was teaching one of my friend how to make beads, now my other friends are now asking me to teach them. I can teach other people what I learned from this program. It is a very program to help me learn this that will help me in the future” (SS2, 17 years).

## Discussion

Although substantial progress has been made towards implementing combination HIV prevention interventions among adolescent girls in school in SSA, few studies have been conducted to assess factors that influence participation in these interventions from the perspectives of participants. To close this gap, the objective of this study was to explore the experiences of the participants and perceived factors that influenced their participation in the intervention. The participants identified several facilitators and barriers to participation in the combination HIV prevention intervention, lending insights to promote intervention reach and adoption.

An understanding of factors that impact intervention participation can provide a nuanced explanation for indicators of intervention reach and adoption. These are vital components to optimize public health intervention (23, 24) and ultimately address the growing disparities of HIV burden among AGYW in SSA.

According to the participants, a key facilitator was the implementation of the intervention components by young female facilitators. Previous HIV prevention studies among AGYW in SSA have highlighted the benefits of female staff delivering intervention components for female participants, thereby enhancing comfort among study participants (25). An intervention study among adolescent girls in South Africa highlighted the benefits of engaging female facilitators for girls-only intervention to create safe spaces for girls to seek help, learn, and build relationships with older women (role models) who have gone through a similar life process (25, 26). These findings support the importance of ensuring that intervention implementers and facilitators are relatable to adolescent girls to promote participation.

Furthermore, receiving the support and approval of participating school authorities was highlighted as another important facilitator. Delivering the intervention in the school environment eased the ability of the participants to obtain permission from their parents/guardians, thus, establishing an increased level of trust and credibility between the school and parents. Our findings suggest that to promote participation in settings where young women are usually under the care of their parents until adulthood, it is important to implement the intervention in a location that parents trust.

Also, the intervention delivery in the school environment was convenient for the young people. It increased the likelihood of attending the intervention, given that it was incorporated in their school activity for the intervention period. This is consistent with previous studies that have highlighted the role of schools in galvanizing skills to promote HIV risk-reduction among young people in SSA (27–29).

Concerning barriers, we uncovered sexual conservatism from society and parents, and challenges in sustaining a microenterprise. Sexual conservatism is a well-documented barrier to HIV intervention participation (30), as it promotes stigma with engaging in sexual activity or seeking sexual and reproductive health services. Discussing topics related to sexual and reproductive health is considered taboo with this mindset (30). Similarly, the participants in our study indicated discomfort with discussing sexual and reproductive health topics in the school environment. Several participants were worried that some of their teachers and students who were not part of the intervention might listen in on the intervention discussions. This finding suggests the need to develop strategies to normalize conversations about sexual and reproductive health. AGYW in Nigeria are disproportionately affected by HIV, of which the primary form of transmission is through heterosexual sex. Therefore, steps should be taken to promote the discussion of these topics in a non-stigmatizing manner. This may help bridge the disconnect between the perception of taboos and the actual reality of increased risk of HIV and other Sexually Transmitted Infections among AGYW in Nigeria.

Regarding challenges in sustaining a microenterprise, the finding suggests that to enhance income-generation activities, it is paramount to assist participants in developing marketplace strategies that attract customers and generate stable income. Research on developing marketplace strategies among individuals, especially women in resource-constrained settings, suggests employing a subsistence marketplace (31, 32). Promoting sustainable marketplace strategies will involve working with AGYW to identify low-cost, culturally appropriate strategies to encourage sales for their products. Future iterations of the intervention may explore the possibility of creating a low-cost in-person and online marketplace for participants to sell their products. This could help address some of the challenges identified by the participants. Also, future studies may explore providing additional income-generating skills to complement the jewelry-making enterprise for participants to engage in other income-generating activities that best suit their needs.

Further assessment of the intervention among study participants provided evidence that the intervention is appropriate and generally acceptable. The participants reported improvement in HIV prevention skills and acquiring skills to share with their family members and peers. Overall, the participants responded positively to the intervention structure and components. The participants were enthusiastic about the interactive and group structure of the intervention. The structure provided a safe space for meaningful interactions with their peers and intervention facilitators. Other interventions with similar structures among AGYW in SSA have had similar findings, with participants articulating the acceptability and appropriateness of intervention components (33–36).

The aims of this study have some limitations that should be noted. As with most qualitative studies, the findings may not be generalizable to other settings. The transferability and applicability of our findings are limited to the participants whom we engaged in the focus group discussions. Also, the study is based on the perspectives of the participants, which may be prone to personal-preferred reporting even with the appropriate probing questions (21, 37, 38). However, the degree of consistency in responses across the focus groups assures and strengthens the credibility of our findings (39). Perceived barriers and facilitators of participation in the intervention were investigated from the perspective of adolescent girls in the intervention, as they are in the best position to share their experience as recipients of the interventions. Given that these young people are often under the guardianship of their school and parents, future studies could explore the perspective of school administrators and parents to gain a more holistic understanding of barriers and facilitators of adolescent participation in school-based interventions. Also, data collection was conducted by the intervention facilitators, which may have influenced their responses to the questions.

This study highlighted barriers to covering some sexual and reproductive health topics among AGYW. Despite these limitations, this study makes several important contributions to the literature. Future research may explore how to tailor an intervention curriculum to optimize for cultural and religious contexts of participants. Also, an important strength of this study was our collection of views from a sizable group of adolescent girls who participated in a combination income-generating HIV prevention intervention. We were able to convene about 65% of individuals who participated in the intervention.

## Conclusions

Despite the perceived benefits and interest in participation in the intervention, the study participants outlined some challenges. Addressing barriers, coupled with the promotion of facilitating factors, is essential for enhancing the engagement of AGYW in HIV risk-reduction intervention. Some modifiable factors were identified, and can be addressed for future studies to improve the experience of participants and potential intervention impact. This implies that combination income-generating HIV prevention interventions aimed at actively engaging adolescent girls should deliver the intervention in settings relatable to the participants. Such intervention should also include strategies to assist participants in generating sustainable income and engage staff who understand the lived experiences and contexts of participants. Our findings can guide future research and design of income-generating HIV prevention intervention for AGYW in low-resource settings such as Nigeria.

## Data Availability Statement

The original contributions presented in the study are included in the article/Supplementary Material, further inquiries can be directed to the corresponding author/s.

## Ethics Statement

Ethical approvals were obtained from the Institutional Review Board at Saint Louis University and the Nigerian Institute of Medical Research. Written informed consent for participation was not provided by the participants' legal guardians/next of kin because: according to the Nigerian guidelines for sexual and reproductive health research, young people who are 13 years and over can provide informed consent for sexual and reproductive health research (40).

## Author Contributions

UN developed the concept of the study and designed the study under the supervision of JI, RB, and JC. UN and WJ assisted with data collection for the study. UN performed the analysis and interpretation. UN wrote the initial draft of the manuscript, which was critically reviewed and revised by TG, WJ, AM, CO, FU, OE, JC, RB, and JI. All authors read and approved the final manuscript.

## Conflict of Interest

The authors declare that the research was conducted in the absence of any commercial or financial relationships that could be construed as a potential conflict of interest.

## Publisher's Note

All claims expressed in this article are solely those of the authors and do not necessarily represent those of their affiliated organizations, or those of the publisher, the editors and the reviewers. Any product that may be evaluated in this article, or claim that may be made by its manufacturer, is not guaranteed or endorsed by the publisher.

## Abbreviations

AGYW: adolescent girls and young women
AM: Adesola Z. Musa
CO: Chisom Obiezu-Umeh
COREQ: consolidated criteria for reporting qualitative research
FU: Florida Uzoaru
JC: Jami Curley
JI: Juliet Iwelunmor
FGD: focus group discussions
IRB: institutional review board
SSA: sub-Saharan Africa
TG: Titi Gbajabiamila
OE: Oliver Ezechi
RB: Rhonda BeLue
UN: Ucheoma Nwaozuru
WT: Wakeela Tijani
YDF: youth development funds.
